# A next-generation bifunctional photosensitizer with improved water-solubility for photodynamic therapy and diagnosis

**DOI:** 10.18632/oncotarget.12366

**Published:** 2016-09-30

**Authors:** Hirotada Nishie, Hiromi Kataoka, Shigenobu Yano, Jun-ichi Kikuchi, Noriyuki Hayashi, Atsushi Narumi, Akihiro Nomoto, Eiji Kubota, Takashi Joh

**Affiliations:** ^1^ Departments of Gastroenterology and Metabolism, Nagoya City University Graduate School of Medical Sciences, Mizuho-cho, Mizuho-ku, Nagoya 467-8601, Japan; ^2^ Graduate School of Materials Science, Nara Institute of Science and Technology, Ikoma, Nara 630-0192, Japan; ^3^ Department of Organic Materials Science, Graduate School of Organic Materials Science, Yamagata University, Yamagata, Yonezawa 992-8510, Japan; ^4^ Department of Applied Chemistry, Graduate School of Engineering, Osaka Prefecture University, Nakaku, Sakai, Osaka 599-8531, Japan

**Keywords:** oligosaccharide-conjugated chlorin, Warburg effect, glycoconjugated chlorin, photodynamic therapy, photodynamic diagnosis

## Abstract

Photodynamic therapy (PDT) exploits light interactions and photosensitizers to induce cytotoxic reactive oxygen species. Photodynamic diagnosis (PDD) uses the phenomenon of photosensitizer emitting fluorescence to distinguish some tumors from normal tissue. The standard photosensitizer used for PDD is 5-aminolevulinic acid (5-ALA), although it is not entirely satisfactory. We previously reported glucose-conjugated chlorin (G-chlorin) as a more effective photosensitizer than another widely used photosensitizer, talaporfin sodium (TS); however, G-chlorin is hydrophobic. We synthesized oligosaccharide-conjugated chlorin (O-chlorin) with improved water-solubility. We report herein on its accumulation and cytotoxicity. O-chlorin was synthesized and examined for solubility. Flow cytometric analysis was performed to evaluate O-chlorin accumulation in cancer cells. To evaluate the intracellular localization of photosensitizer, cells were stained with O-chlorin and organelle-specific fluorescent probes. We then measured the *in vitro* fluorescence of various photosensitizers and the half-maximal inhibitory concentrations to evaluate effects in PDD and PDT, respectively. Xenograft tumor models were established, and antitumor and visibility effects were analyzed. O-chlorin was first shown to be hydrophilic. Flow cytometry then revealed a 20- to 40-times higher accumulation of O-chlorin in cancer cells than of TS, and a 7- to 23-times greater fluorescence than 5-ALA. *In vitro*, the cytotoxicity of O-chlorin PDT was stronger than that of TS PDT, and O-chlorin tended to accumulate in lysosomes. *In vivo*, O-chlorin showed the best effect in PDT and PDD compared to other photosensitizers.

O-chlorin was hydrophilic and showed excellent tumor accumulation and fluorescence. O-chlorin is promising as a next-generation bifunctional photosensitizer candidate for both PDT and PDD.

## INTRODUCTION

Photosensitizers are molecules that undergo photochemical reactions in response to specific light irradiation to emit fluorescence. Photodynamic therapy (PDT) exploits this phenomenon to generate reactive oxygen species (ROS) such as singlet oxygen [1, 2], and is an established treatment for cancer and some nonmalignant diseases [2–6]. PDT has several advantage over conventional cancer treatment; it is relatively noninvasive and causes less systemic toxicity [3]. Furthermore, the same photosensitizers can also be used for photodynamic diagnosis (PDD) based on the emission of fluorescence when irradiated in the presence of specific abnormal cells. The antitumor mechanism of PDT manifests as three patterns. The first is a direct toxicity action due to ROS generation inside the tumor; the second is a shutdown effect induced by starving the surrounding vessels, leading to tumor infarction; and, the third is a normal immunological mechanism activated via the tumor response to PDT [1, 2, 7, 8]. The photosensitizer, 5-aminolevulinic acid (5-ALA), which is converted at the tissue level to its active compound protoporphyrin IX, has been used historically for PDD in many medical fields because it accumulates strongly in tumor cells rather than in normal tissue due to their difference cellular metabolisms [9–13].

Since PDT and PDD were introduced more than a quarter of a century ago, various improvements have been attempted for better clinical results, including coordination of photochemical wavelengths, developments in laser generation, improved drug delivery systems, and of course, new and improved photosensitizer chemicals [14]. In this context, we previously reported a glucose-conjugated chlorin compound (G-chlorin) as a more effective photosensitizer than the conventional ones used thus far [15, 16], based on a phenomenon called the Warburg effects whereby cancer cells in general consume more glucose than normal cells [17]. Subsequently, we developed a mannose-conjugated chlorin (M-chlorin), which proved more efficient than G-chlorin in generating anti-tumor cytotoxicity and in suppressing tumor-associated macrophages [18]. However, when these glycoconjugated chlorins were administered intravenously, the reagents had to be dissolved into organic solvent due to their hydrophobicity. In recent years, Talaporfin sodium (TS), a second-generation photosensitizer in Japan, has been widely taken up for clinical use due to its improved water solubility and excretion [19, 20]; however, TS has not yet replaced the ‘gold standard’ for PDT. Furthermore, in the field of PDD, almost all reports cite 5-ALA because it specifically visualizes some tumors and has few side effects [9, 10, 13, 21, 22].

We recently succeeded in isolation of a novel glycoconjugated compound, hydrophilic O-chlorin having four maltotriose units [23], In this study we evaluated its cancer-selective accumulation, discrimination, and cytotoxicity as a bifunctional photosensitizer for both PDT and PDD.

## RESULTS

### Newly synthesized O-chlorin showed water solubility

**Figure 1:**
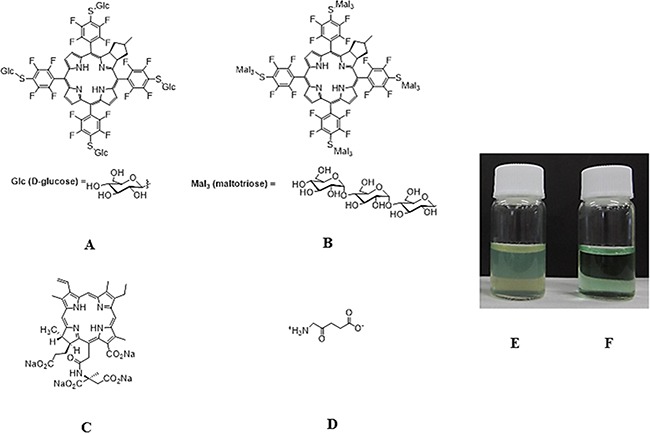
Chemical structure of G-chlorin, O-chlorin, TS, and 5-ALA and water solubility of O-chlorin **A.** Glucose-conjugated chlorin (G-chlorin); 5, 10, 15, 20- tetrakis (4- (β- D- glucopyranosylthio)- 2, 3, 5, 6- tetrafluorophenyl)- 2, 3- (methano (*N*-methyl) iminomethano) chlorin. **B.** oligosaccharide(maltotrisose)-conjugated chlorin (O-chlorin); 5, 10, 15, 20- tetrakis (4- (β- D- maltotriosylthio)- 2, 3, 5, 6- tetrafluorophenyl)- 2, 3- (methano (*N*-methyl) iminomethano) chlorin. **C.** talaporfin sodium; (mono-l-aspartyl chlorin6) Laserphyrin®. **D.** 5-ALA (5-Aminolevulinic acid). (A; G-chlorin, B: O-chlorin, C: TS, D: 5-ALA). G-chlorin solution was opaque due to its water insolubility, whereas O-chlorin was clear. (**E.** G-chlorin solution, **F.** O-chlorin solution).

We recently synthesized a glycoconjugated chlorin named O-chlorin that contains glucose-derived oligosaccharides, such as maltotriose. Compared to G-chlorin, O-chlorin proved to be more highly water-soluble (Figure 1E, 1F) [23]. This high solubility enabled O-chlorin to be administered intravenously and to reach the whole body without addition of a toxic organic solvent.

### The accumulation of O-chlorin in cancer cells was much higher than that of TS

**Figure 2:**
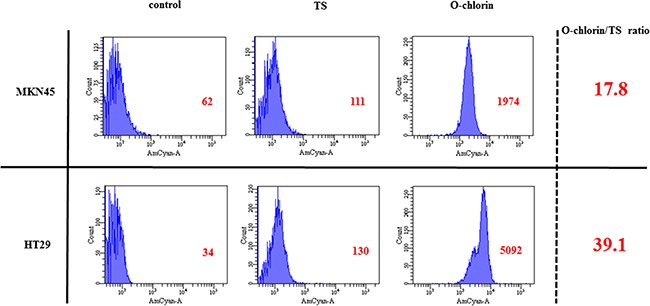
The accumulation of O-chlorin and TS in cancer cells MKN45 and HT29 were loaded with O-chlorin, TS, or no reagent as a control for 4 h, and then analyzed for cell accumulation using flow cytometry at 405nm excitation and 680 nm emission. O-chlorin and TS both contained chlorin as a photosensitizer, with nearly peak excitation and emission wavelengths reached for the chlorin. The abscissa of the graph indicates populations of cells and the ordinate represents the intensity of emission. Red figures show mean areas.

We first examined the uptake of TS and O-chlorin *in vitro* using MKN45 and HT29 cells. Cells were incubated with 5 μM photosensitizer for 4 hours, and uptake was estimated by measuring the intensity of the characteristic red fluorescence at the single cell level using FACS. The accumulation of O-chlorin in both cancer cells was 20-40 times higher than that of TS (Figure 2).

### O-chlorin accumulated in cancer cells and was mainly localized in lysosomes

**Figure 3:**
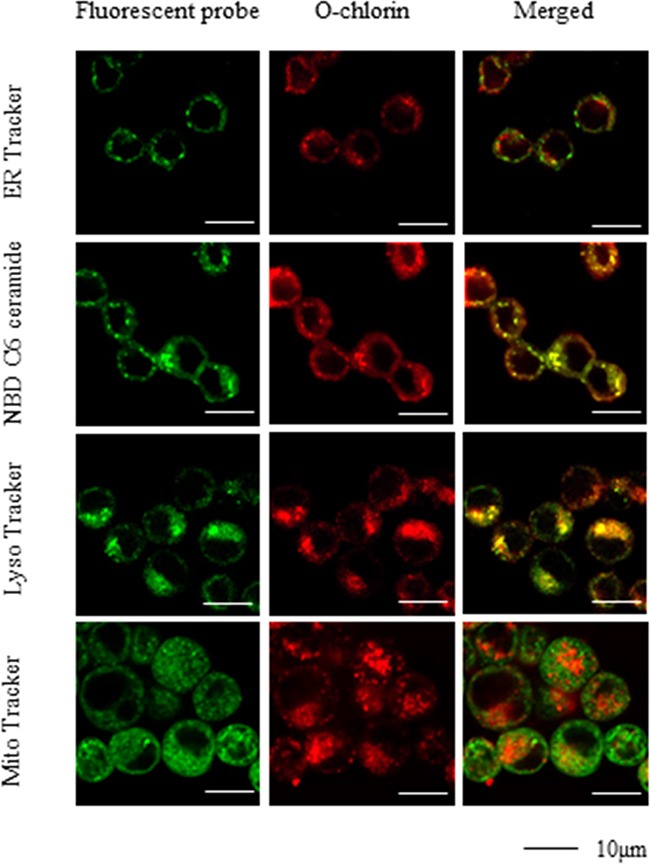
Subcellular localization of O-chlorin MKN45 cells were loaded with O-chlorin for 24 hours and labeled with Mito Tracker Green, Lyso Tracker Green, NBD C6 ceramide Green, or ER Tracker Green. The images were obtained by confocal microscopy (Original magnification, ×300; scale bar, 10 μm).

We tested the accumulation and subcellular localization of O-chlorin by confocal microscopy using fluorescence probes to mark intracellular organelles. Cells were loaded with O-chlorin and incubated with MitoTracker Green, LysoTracker Green, NBD C6 ceramide Green, or ERTracker Green to label mitochondria, lysosomes, Golgi, or endoplasmic reticula, respectively. The detection of O-chlorin tended to coincide with LysoTracker, indicating accumulation in lysosomes (Figure 3).

### The fluorescent activity of O-chlorin exceeded that of 5-ALA

**Figure 4:**
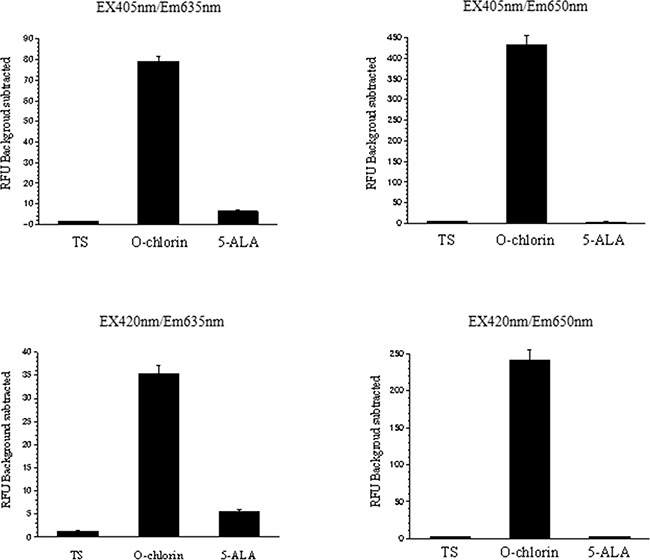
Fluorescence of photosensitizers in MKN45 cells under various irradiations of LED light MKN45 cells were incubated with TS, O-chlorin, or 5-ALA, then fluorescence intensities were measured using a microplate reader. Light was irradiated under condition of 405 nm and 420 nm excitation, and 635 nm and 650 nm emission. Data are means ± SE of eight independent experiments.

We next evaluated the PDD ability *in vitro* by measuring fluorescence of TS, O-chlorin, and 5-ALA. MKN45 cells were incubated with TS, O-chlorin, or 5-ALA, and then analyzed by microplate reader to measure the fluorescence of each reagent. The intensity of O-chlorin fluorescence was strongest, followed by 5-ALA, and then TS under the condition of 405 nm and 420 nm excitation and 635 nm and 650 nm emission (Figure 4). In various cancer cell lines, the fluorescence of O-chlorin was 7 to 23 times stronger than that of 5-ALA (Table 1).

**Table 1:** The effect of PDD among TS, 5-ALA and O-chlorin in various cell lines

Photosensitizer				
Cell line	TS	5-ALA	O-chlorin	O-chlorin/5-ALA ratio
MKN45	1. 97 ± 0. 20	11. 38 ± 1. 93	80. 26 ± 2. 77	7. 05
HT29	1. 52 ± 0. 10	6. 31 ± 0. 67	79. 17 ± 2. 40	12. 55
OE21	2. 39 ± 0. 11	8. 04 ± 0. 55	76. 78 ± 3. 40	9. 55
KYSE30	2. 92 ± 0. 14	5. 26 ± 0. 41	123. 09 ± 2. 54	23. 40

### PDD with O-chlorin clearly identified tumors *in vivo*

**Figure 5:**
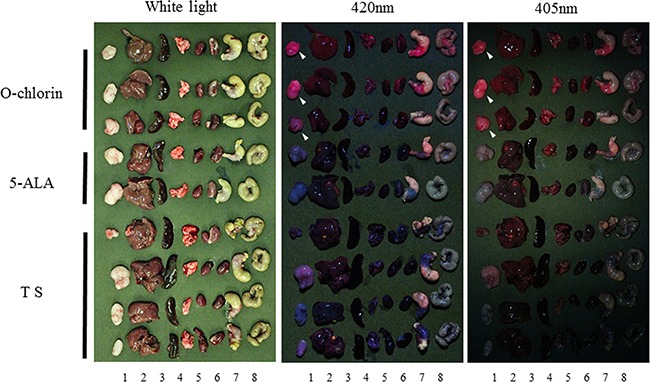
PDD effect in xenograft tumor model Once tumors reached a sufficient size, mice were administered O-chlorin, 5-ALA, or TS at a dose of 2.5 μmol/kg, 50 μmol/kg, or 2.5 μmol/kg, respectively. 4 hours after administration, mice were sacrificed, and tumor and organs were extracted. The excised tissues were observed under irradiation by white light and LED light of 405 nm and 420 nm. The white arrowhead indicates tumors administered with O-chlorin and showing strong fluorescence. Sample number of O-chlorin, 5-ALA, and TS were three, two, and four, respectively. Numeral below the figure indicates tumor and organs (1; tumor, 2; liver, 3; spleen, 4; lung, 5; heart, 6; kidney, 7; stomach, 8; colon).

We then investigated the photosensitizer fluorescence in a xenograft tumor mice model. Once the implanted tumors reached a sufficient size, O-chlorin, 5-ALA, or TS were administered to mice at a dose of 2.5 μmol/kg, 50 μmol/kg, or 2.5 μmol/kg, respectively, and all mice were sacrificed 4 hours later. Tumor and various organs were then extracted and observed under white light and LED light irradiation. No difference was seen among the photosensitizers under white light; however, under 405 nm and 420 nm LED light, tumors administered with O-chlorin clearly emitted red fluorescence, those receiving TS or 5-ALA emitted little. Furthermore, there was little or no emission from the non-tumor organs for all reagents (Figure 5).

### PDT with O-chlorin showed higher cytotoxicity than that with TS in esophageal, gastric, and colon cancer cells

To evaluate the effectiveness of PDT with O-chlorin, we evaluated the cell death induced by PDT using O-chlorin. Cells were loaded with TS or O-chlorin for 24 hours, irradiated with 660-nm red LED light at 16 J/cm^2^, and incubated for 24 hours. We performed WST assays to determine the IC_50_ at 24 hours after irradiation. As shown in Table 2, PDT using O-chlorin induced cell death with 7 to 50 times higher cytotoxicity than TS in all cancer cells.

**Table 2:** Comparison of 50% inhibition concentration (IC_50_) by PDT between TS and O-chlorin

	Esophageal cancer		Gastric cancer		Colon cancer
	OE21	KYSE30	MKN28	MKN45	HT29
TS	17.4	4.29	12.7	11.4	18.55
O-chlorin	0.33	0.11	0.56	1.25	0.97

### O-chlorin PDT suppressed tumor growth strongly *in vivo*

**Figure 6:**
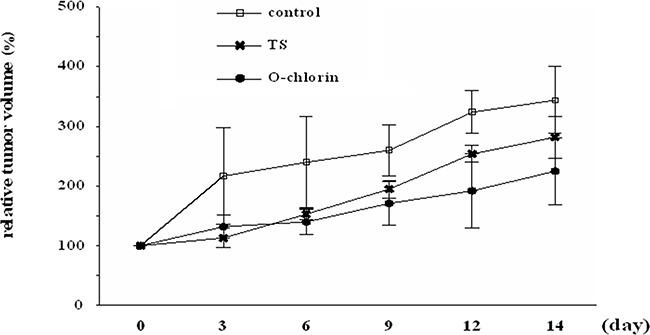
Antitumor effects of PDT in mouse xenograft model Mice were irradiated with 15 J/cm^2^ of diode laser at 664 nm 4 hours after injection of the photosensitizer. PDT was performed on day 0 and tumor volumes were monitored for 14 days in total. Data are shown as ± SE (n = 3 for control, n = 6 for TS and O-chlorin).

We finally evaluated the antitumor effects of O-chlorin PDT on xenograft tumors in mice. 14 days after tumor inoculation, mice were administered O-chlorin or TS intravenously at a dose of 0.625 μmol/kg or 6.25 μmol/kg, respectively. After 4 hours, tumors ware irradiated with the 664-nm LASAR at 15 J/cm^2^. The PDT using O-chlorin strongly suppressed tumor growth compared to TS, even at one-tenth the amount of TS (Figure 6).

## DISCUSSION

In this study we proved that newly developed O-chlorin PDT was superior to TS PDT, the second-generation PDT widely used in Japan, due to increased tumor accumulation and cytotoxicity. We previously reported that conjugating sugar chains onto photosensitizer compounds increases their cellular uptake and antitumor effect, thus glycoconjugated chlorin reagents should achieve more effective PDT than TS PDT. While G-chlorin and M-Chlorin proved to be water insoluble [15, 16, 18], the recently synthesized O-chlorin has higher water-solubility by virtue of more attached sugar chains than G-chlorin (Figure 1E, 1F) [23]. For PDD, O-chlorin showed higher tumor accumulation than 5-ALA, the long-held ‘gold standard’ in clinical use worldwide for PDD.

With respect to the concentration of photosensitizer needed for PDT, Allison et al. [7] recommended using as little photosensitizer as possible to achieve a sufficient anti-tumor response. The higher cellular uptake and accumulation shown by O-chlorin *in vitro* and *in vivo* compared to TS should therefore enable lower amounts of photosensitizer to be used clinically for PDT. Such an outcome would also reduce associated adverse events like skin disorders. Indeed, in this study, the half-maximal inhibitory concentration (IC_50_) of O-chlorin in cancer cells was 11-59 times lower than that of TS. PDT effects on patients also vary according to the localization of photosensitizer accumulation. ROS generated via PDT have a short half-life and acts close to their site of generation. Consequently, the type of photodamage to cells loaded with a photosensitizer could depend on its precise subcellular localization [8]. Herein, we localized O-chlorin mainly in the lysosomes of cancer cells, at sites proposed to be critical for photosensitizer action [24]. However, later studies reported that although lysosomally localized photosensitizers can lead to cell killing upon illumination, the relative efficacy is significantly lower than that seen with photosensitizers localized in mitochondria and other organelles [25]. Mitochondrial damage after illumination is a particularly important mechanism of apoptotic cell death induced via PDT [26]. Nagata et al. [27] also showed lysosomes as the primary site of chlorin-based photosensitizer accumulation, and that these cells underwent apoptosis upon irradiation doses leading to 70% cell death, suggesting that apoptotic pathways are activated via mitochondrial destabilization following PDT damage to lysosomes. Thus, the preferential accumulation of O-chlorin in cancer cell lysosomes in our studies could have induced apoptosis via lysosomal damage even with low doses of photosensitizer.

The characteristics of an ideal photosensitizer have been discussed in recent reviews and indicated important factors, including good water-solubility [28, 29]. O-chlorin would therefore be an ideal photosensitizer, with almost the same antitumor effect as G-chlorin and the same hydrophilicity as TS. For PDD, O-chlorin showed higher fluorescence activity in various cancer cell lines *in vitro* than 5-ALA, conventionally used worldwide. Moreover, the xenograft tumors dosed with O-chlorin clearly emitted fluorescence *in vivo*, while those with TS and 5-ALA emitted little (Figure 4). For over 25 years, PDD using 5-ALA was used in various fields such as neurosurgery, urology, gastroenterology, and gynecology. Especially in neurosurgery and urology, PDD has an increasingly important role in deciding surgical margins prior to and during surgery, resulting in improved prognoses [9–13, 30, 31]. Based on this study, O-chlorin has prospective value as a novel and conventional PDD reagent, to detect and discriminate various tumor locations.

In terms of mechanism, we speculate that the major antitumor effect of O-chlorin PDT could be ROS-induced direct toxicity, as for TS. PDT using TS also works by tumor shutdown, and our *in vivo* examinations implicated this mechanism for O-chlorin PDT because TS and O-chlorin have the same chemical structure. The precise mechanism of O-chlorin PDT remains to be clarified.

In conclusion, we synthesized O-chlorin, which showed good water-solubility, strong accumulation to tumors, and high antitumor efficacy, suggesting it to be an efficient and tolerable reagent for PDT and PDD. Finally, O-chlorin has temporal and economic benefits by enabling PDT and PDD to be performed using a single photosensitizer and at the same time. Therefore, O-chlorin is a novel candidate for the ideal next-generation “bifunctional” photosensitizer.

## MATERIALS AND METHODS

### Photosensitizers

The photosensitizer compounds, O-chlorin (TFPC–SMal_3_) 5,10,15,20-tetrakis-[4-(β-D-maltotriosylthio)-2,3,5,6-tetrafluorophenyl]-2,3-[methano- (N-methyl) iminomethano]chlorin and G-chlorin (H_2_TFPC-SGlc)(5,10,15,20-tetrakis(pentafluorophenyl)-2,3-(methano[N-methyl]iminomethano])chlorin) were synthesized and provided by laboratories at Yamagata University (Japan) and Nara Institute of Science and Technology (Japan) (Figure 1A, 1B). TS (mono-l-aspartyl chlorin6, Laserphyrin®) was purchased from Meiji Seika (Tokyo, Japan) (Figure 1C), and 5-ALA was purchased from Cosmo Bio Co., LTD (Tokyo, Japan) (Figure 1D).

### Cell culture

The human esophageal cancer cell line OE21 (No.11D028; ECACC) was cultured in RPMI 1640 medium (Wako Pure Chemical Industries) while KYSE30 was grown in a 50/50 media mix of RPMI 1640 and Ham's F12 (Wako Pure Chemical Industries) supplemented with 2 mM glutamine, 2% fetal bovine serum (FBS), 100 U/mL penicillin, 100 mg/mL streptomycin, and 0.25 mg/mL amphotericin B. The HT29 colon cancer cell line (No. HTB-38; ATCC) was cultured in McCoy's 5A medium (Life Technologies) supplemented with 10% FBS and 100 U/mL penicillin, 100 mg/mL streptomycin, and 0.25 mg/mL amphotericin B. The MKN45 human gastric cancer cell line (No. 0254; Japanese Cancer Research Bank) was cultured in RPMI 1640 supplemented with 10% FBS and 100 U/mL penicillin, 100 mg/mL streptomycin, and 0.25 mg/mL amphotericin B. Cells were cultured under an atmosphere of 5% CO_2_ at 37°C.

### Animals and tumor models

Female nude mice (BALB/c Slc-nu/nu) of 4-6 weeks old and weighing15-20 g were purchased from Japan SLC. Before initiating any interventions, mice were allowed to acclimatize for at least 2 weeks in the animal facility. Xenograft tumor models were established by subcutaneously implanting 1 × 10^6^ MKN45 cells in 100 mL of culture media under the right flank of experimental mice. The procedures and experiments were approved by Nagoya City University Center for Experimental Animal Science, and mice were cared for according to the guidelines of the Nagoya City University for Animal Experiments.

### Flow cytometric analysis

Cancer cells were seeded into 6-cm culture dishes at 2 × 10^5^ cells/well and incubated at 37°C for 48 hours. After removing the medium to evaluate the accumulation of photosensitizer into cells, fresh medium supplemented with 5 μM photosensitizer were added to the dishes for 4 hours. Cells were then washed with phosphate-buffered saline (PBS) three times and removed from the culture dish with TrypLE-Express (Invitrogen) for analysis using a FACSCant II (BD Biosciences) at excitation and emission wavelengths of 405 nm and 680 nm, respectively. At least 10,000 events were collected for each sample.

### Intracellular localization of photosensitizers

MKN45 cells were seeded onto coverslips placed in 12-well culture plates at 1 × 10^5^ cells/well and incubated for 24 hours. Subsequently, 1 μmol/L O-chlorin was added to the culture media and the cells were incubated for a further 4 hours before staining with organelle-specific fluorescent probes. Lysosomes were stained with 0.1 μmol/L LysoTracker Green (Invitrogen) at 37°C for 30 minutes, mitochondria with 0. 1 μmol/L MitoTracker Green FM (Invitrogen) at 37°C for 10 minutes, Golgi with 5 μmol/L NBD C6-ceramide at 4 °C for 30 minutes, and endoplasmic reticulum with 0.1 μmol/L ER-Tracker Green (Invitrogen) at 37°C for 30 minutes. After incubation, culture media were replaced with fresh medium to remove free dyes, and then the stained cells for observed, live for mitochondria and following fixation with 4% paraformaldehyde for lysosomes, Golgi, and endoplasmic reticulum. To visualize the localization, confocal laser microscopy (Nikon A1 confocal system Nikon Instech Co., Ltd.) was used and the obtained data were analyzed with NIS element imaging software (Nikon). Band-pass emission filters of 505-530 nm and 650 nm were used.

### Fluorescence of photosensitizers *in vitro*

The effect of PDD *in vitro* was evaluated by measuring fluorescence in the cancer cell lines. The esophageal, gastric, and colon cancer cells (OE21, KYSE30, MKN45, HT29) were seeded in opaque 96-well culture plates at 5 × 10^3^ cells/100 mL/well and incubated for 24 hours. The media were removed and the cells were incubated for a further 4 hours in medium supplemented with TS, 5-ALA, or O-chlorin at 5 μmol/L, 1 mmol/L, and 5 μmol/L, respectively. Then, cells were washed three times with PBS and covered left in PBS until the fluorescence of each reagent was measured using a microplate reader (Gemini EM, Molecular Devices). Intensity of fluorescence was expressed as a relative fluorescence unit background subtracted. The obtained data were analyzed with SoftMAX pro software (Molecular Devices). The wavelengths of excitation used for analysis were 405 nm and 420 nm, and for emission were 635 nm and 650 nm, with data collected from eight independent experiments.

### *In vivo* PDD

After the tumor implanted on mice reached approximately 100mm^3^, solution of TS, 5-ALA, or O-chlorin was injected via the tail vein at a dose of 2.5μmol/kg, 50μmol/kg and 2.5μmol/kg, respectively. After 4 hours, mice were sacrificed according to our institutional guidelines followed by extraction of tumor and organs including liver, spleen, lung, heart, kidney, stomach, and colon. Subsequently, tumor and organs were observed under white light and under 405 nm and 420 nm irradiation of LED light. The image was obtained using a high resolution camera equipped with optical filter (cut-on:470 nm, Longpass Filter/VIS 470nm, Asahi Spectra Co., Ltd).

### *In vitro* PDT

The esophageal, gastric, and colon cancer cells (OE21, KYSE30, MKN45, HT29) were incubated with photosensitizer in culture medium. After 24 hours, cancer cells were washed once with PBS, covered with PBS, and irradiated with LED light (Optocode corporation) which emits 660 nm wavelength at an energy of 16 J/cm^2^ (intensity: 36 mW/cm^2^).

### Cell viability assay

Cell viability was evaluated by the WST-8 cell proliferation assay (Dojindo). Cancer cells were seeded in 96-well culture plates at 5 × 10^3^ cells/100 mL/well and incubated overnight. Subsequently, cells were incubated with TS or O-chlorin at 37°C for 24 hours, irradiated, and then incubated with culture medium for a further 24 hours. Cells were incubated with the cell counting kit-8 solution for 2 hours and absorption at 450 nm was measured using a microplate reader (SPECTRA MAX340, Molecular Devices). Cell viability was expressed as a percentage of untreated control cells. The half-maximal (50%) inhibitory concentration (IC_50_) was calculated for each reagent.

### *In vivo* PDT

When the implanted tumors grew to approximately 100 mm^3^, mice were divided into three groups with comparable average tumor volumes. Subsequently, O-chlorin or TS was administered to mice in the appropriate groups via tail vein injection at a dose of 0.625 μmol/kg or 6.25 μmol/kg, respectively. Four hours after administration, the tumors were irradiated using a 664-nm red LASER (OK Fiber Technology) at a dose of 15 J/cm^2^ (intensity: 150mW/cm^2^) applied to the skin directly above the tumors. Treatment was performed only once at 7 to10 days after tumor inoculation. Tumor growth was monitored once every three days by measuring the tumor volume with Vernier calipers and the tumor volume was calculated by the formula, length × width × depth/2. The results were analyzed using the Bonferroni–Holm method to assess differences between groups.
